# An Enhanced Ensemble Approach for Non-Intrusive Energy Use Monitoring Based on Multidimensional Heterogeneity

**DOI:** 10.3390/s21227750

**Published:** 2021-11-21

**Authors:** Yu Liu, Qianyun Shi, Yan Wang, Xin Zhao, Shan Gao, Xueliang Huang

**Affiliations:** 1 School of Electrical Engineering, Southeast University, Nanjing 210018, China; 220203012@seu.edu.cn (Q.S.); 220202976@seu.edu.cn (Y.W.); xinzhao@seu.edu.cn (X.Z.); shangao@seu.edu.cn (S.G.); xlhuang@seu.edu.cn (X.H.); 2 Jiangsu Provincial Key Laboratory of Smart Grid Technology and Equipment, Nanjing 210018, China

**Keywords:** artificial intelligence, energy disaggregation, ensemble method, heterogeneous design, non-intrusive load monitoring

## Abstract

Acting as a virtual sensor network for household appliance energy use monitoring, non-intrusive load monitoring is emerging as the technical basis for refined electricity analysis as well as home energy management. Aiming for robust and reliable monitoring, the ensemble approach has been expected in load disaggregation, but the obstacles of design difficulty and computational inefficiency still exist. To address this, an ensemble design integrated with multi-heterogeneity is proposed for non-intrusive energy use disaggregation in this paper. Firstly, the idea of utilizing a heterogeneous design is presented, and the corresponding ensemble framework for load disaggregation is established. Then, a sparse coding model is allocated for individual classifiers, and the combined classifier is diversified by introducing different distance and similarity measures without consideration of sparsity, forming mutually heterogeneous classifiers. Lastly, a multiple-evaluations-based decision process is fine-tuned following the interactions of multi-heterogeneous committees, and finally deployed as the decision maker. Through verifications on both a low-voltage network simulator and a field measurement dataset, the proposed approach is demonstrated to be effective in enhancing load disaggregation performance robustly. By appropriately introducing the heterogeneous design into the ensemble approach, load monitoring improvements are observed with reduced computational burden, which stimulates research enthusiasm in investigating valid ensemble strategies for practical non-intrusive load monitoring implementations.

## 1. Introduction

Knowing the refined electricity behaviors of household energy consumption is important to residents, by means of which energy consciousness can be awakened and energy conservation schemes can be customized [1]. Meanwhile, it is also important to power utilities, where understanding the load components helps to model power system operations and schedule the demand response better [2]. Furthermore, it is also meaningful to the development of the entire power industry, e.g., it is the technological base of tracking household energy carbon emissions [3]. Therefore, insights into household electricity usage are emerging as a vital link in the energy consumption chain and are attracting more and more attention in both academic and industrial fields.

A straightforward way to realize refined electricity monitoring is to install smart sockets for target appliances and form a sensor network for household electricity monitoring [4]. The exploration enthusiasm for such a project had lasted for a period of time, but it decreased due to the high financial costs associated with too many sockets [5]. Besides, this method of electricity monitoring is strictly confined to socketed appliances, and it is not friendly to residents due to intrusive installations [6]. Hence, the socket-based sensor network, considered as the intrusive way, was replaced once non-intrusive approaches achieved reliable performance.

Non-intrusive load monitoring, NILM for short, is a technology proposed by Professor Hart from MIT. The key idea of NILM is to use a disaggregation algorithm instead of sensor hardware to realize individual appliance monitoring [7]. Thus, the original electric topology and measurements do not need to be changed, and only the service panel data are required, which can be captured by the existing electric meter [8]. Such non-intrusive technology obviously decreases the monitoring cost, covers all appliances, and furthermore does not interfere with residents’ normal life. Therefore, it is widely accepted, and receives much attention in the field of household monitoring.

It is certainly the case that, to realize all the above advantages of NILM, the applied disaggregation approach should be reliable in the first place. Although proposed in 1990s, NILM has emerged as a potential solution only in recent years due to the development of artificial intelligence technologies [9]. Acting as the key pillar during the early development stage of artificial intelligence, pattern recognition plays an important role in the field of non-intrusive load monitoring. Classification technologies, which reflect the essence of pattern recognition, have been widely investigated for NILM research. Considering different appliance characteristics, diverse electric features can be utilized for classification in NILM, such as wavelet-based classification [10] and event-based classification [11]. As to the classification algorithms, the classic k-nearest neighbors was explored in [12] to improve the accuracy and efficiency of NILM. Further on, a support vector machine has been introduced in an early stage [13], enhancing the classification of five nearest neighbor methods. Besides, a novel neuro-fuzzy classification approach is proposed in [14] to address the uncertainties in NILM. Furthermore, multi-label classification was proposed in [15] as a solution with the highest potential for NILM problems, and was widely discussed in the following years [16,17]. In addition to classification, other pattern recognition algorithms also draw attention in the field of load disaggregation field. For example, non-negative matrix factorization has been discussed in various works and demonstrated to be effective in revealing the hidden pattern of energy consumption monitoring, which is suitable for the non-intrusive load disaggregation of large buildings, e.g., industrial buildings [18] and hospital buildings [19]. A revised form of matrix factorization, namely independent-variation matrix factorization, was proposed in [20] to recover positive sources with strong temporal dependency and independent variations, achieving a feasible NILM solution for commercial buildings.

As seen, pattern recognition approaches have already drawn wide attention and achieved considerable results in the field of NILM. However, some limitations show up as the research goes further, e.g., the recognition performance is highly dependent on the expert featured model. Such drawbacks are also observed along with the development of artificial intelligence; therefore, machine learning was developed as an effective alternative [21,22]. Because pattern recognition and machine learning are both implementation methods of artificial intelligence, and machine learning was developed on the basis of pattern recognition, all the approaches discussed above can be categorized into machine learning. In non-intrusive monitoring problems, machine learning is highlighted due to strong self-learning ability, such as semi-supervised learning [23] and unsupervised learning [24]. As a representative, clustering shows a good performance in NILM studies, while k-means clustering is able to deal with unlabeled appliances [25] and density peak clustering improves the disaggregation remarkably [26]. Similarly, as an important branch in machine learning, neural networks show reliable performance in NILM, where convolutional neural networks [27], recurrent neural networks [28], and Siamese neural networks [29] are all demonstrated to be effective. In particular, deep neural networks can be improved for NILM by embedding a denoising autoencoder scheme, which is a new trend in NILM research [30]. As the research progresses, researchers also find that dictionary learning models, which are another machine learning approach, exactly match the key idea of load disaggregation [31]. Showing potential in NILM implementations, dictionary learning was proven to be an outstanding formulation, naturally applicable to NILM [32]. Additionally, further inspired by the sparse coding principle of dictionary learning, transform learning was also explored in [33] and was found to be well-adapted to NILM formulation.

In recent years, the practical experiences from world-leading artificial intelligence races show that the ensemble method is the most powerful approach in machine learning. Therefore, although limited, researchers have noticed the value of ensemble methods in NILM studies, and conducted some explorations. In [34], a multiscale wavelet packet tree is applied to collect comprehensive energy consumption features, and an ensemble bagging tree is adopted as a classifier, where the performance is compared with various machine learning schemes. In [35], an event detection and disaggregation framework based on an ensemble approach is proposed, whose disaggregation target is the water heating operation. Both of the above works focus on event-based load monitoring. Our team has established a general ensemble framework based on bagging in [36] for the load disaggregation of steady-state data, and proved its performance robustness and model flexibility in diverse NILM scenarios.

However, to our knowledge, the current ensemble strategies applied to NILM all follow the evaluation criterion used in individual classifiers, even the probabilistic quantitative scoring method proposed in [36]. Such implementation requires the individual classifier to be reliable and differentiated, but the bias can hardly be avoided since the combined classifier and individual classifiers are homogeneous. If the classifiers are chosen in an inappropriate way, e.g., overemphasizing a specific electrical feature, some errors may be generated and finally cause false decomposition. Since the combined classifier needs the information from individual classifiers for decision making, such disadvantages always exist in the ensemble decision system with homogeneous classifiers, only explicitly or implicitly. Based on this observation and knowledge, the idea of utilizing a heterogeneous design for ensemble-approach-based NILM is proposed and investigated in this paper. Firstly, the multidimensional heterogeneity for an NILM-oriented ensemble method is discussed. Since the individual classifiers can be naturally distinct in a traditional ensemble framework, our research is featured by investigating the heterogeneities from the following aspects, i.e., the heterogeneity between the combined classifier and individual classifiers, as well as the heterogeneity in independent evaluation committees of the combined classifier. Then, an implementation design is illustrated, where the individual classifiers are established based on dictionary learning, while the sparsity is not considered in the combined classifier. Meanwhile, multiple committees with distinguishing similarity measures are employed and coordinated in the decision-making stage, providing valid disaggregation evaluations from multi-perspective points. Through verifications on both a simulation platform and a field measurement dataset, the proposed idea and strategy are proven to be effective in enhancing NILM performance.

The major contribution of this paper is the presence of a multidimensional-heterogeneity-enhanced ensemble approach for NILM. By introducing heterogeneity, the obstacles of ensemble application, including design difficulty and computational inefficiency, are overcome. In addition to providing an effective method to improve NILM performance, this study also stimulates the explorations of applying the ensemble method to NILM for robust and reliable disaggregation. Furthermore, deep thinking of the nature of NILM problems as well as the rationality and completeness of disaggregation models is also inspired. In support of the contribution, the following aspects are highlighted:

- Based on the properties of NILM problems, the heterogeneous evaluation design is utilized in an ensemble model.
- Dictionary learning is deployed for basic load disaggregation, while the sparsity measures are featured in individual classifiers.
- The combined classifier is free of sparsity measures, but composed of multiple decision committees with different similarity measures.
- Verifications on both a simulation platform and a field measurement dataset show the effectiveness of our work.

## 2. Methodology

The task of non-intrusive load monitoring is to disaggregate the detailed appliances’ states via integral electrical measurements. In other words, it is to distinguish the components of monitoring signals, which can be formulated as:

(1) $$x=\sum x_{i},i\in \Omega$$

where ***x*** ∈ ***R****^S^*^×1^ is the target signal with the length of *S*, ***x****_i_* ∈ ***R****^S^*^×1^ is the *i*th appliance selective electrical signature in length, *S*, and Ω stands for the candidate appliance set.

Considering the background noise and signature fluctuations, Equation (1) can hardly be followed in practical applications. Therefore, an error term is usually considered in load disaggregation problems, i.e.,:

(2) $$x=\sum x_{i}+e_{0},i\in \Omega$$

where ***e***_0_ ∈ ***R****^S^*^×1^ is the error term for the decomposition and also in length, *S*.

Since background noise always exists in daily power consumption, and appliances’ operation states are highly dependent on manufacturing standards and electrical aging, the error term provided in Equation (2) not only exists but also plays a key role in load decomposition. The objective of the multidimensional-heterogeneity-enhanced ensemble model is to evaluate the error term from diverse perspectives and avoid the bias caused by certain evaluation approaches.

### 2.1. Ensemble Method Framework

**Figure 1:**
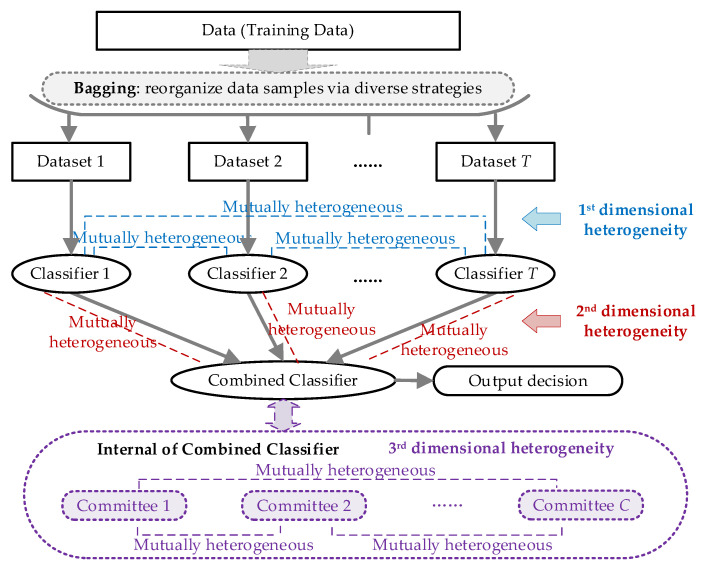
Multidimensional-heterogeneity-enhanced ensemble framework for NILM.

Following the load disaggregation formulation provided in Equation (2), the corresponding ensemble method framework is established in Figure 1, while the proposed idea of multidimensional heterogeneity enhancement is highlighted in color.

As seen in Figure 1, the NILM-oriented ensemble framework follows the bagging strategy. The proposed multidimensional heterogeneity is integrated in the architecture with the following considerations:

1. First: Dimensional heterogeneity in the individual classifiers. The basic idea of bagging is to establish several weak classifiers to combine into a strong classifier. For an effective combination, the weak classifiers should be distinctive from each other. Therefore, the individual classifiers may be heterogeneous according to the definition of the ensemble method. Therefore, in our following sections we do not present the detailed discussions of this point. However, considering the entirety of the description, we still illustrate this dimension in Figure 1 for readers to understand it better.
2. Second: Dimensional heterogeneity between the combined classifier and individual classifiers. The individual classifiers act as the basic appliance disaggregation tool in ensemble-method-based NILM, and the combined classifier acts as the ultimate decision maker. Therefore, if these classifiers are homogeneous, the disaggregation results may be biased, following the features of the applied algorithms. Hence, we introduce heterogeneous evaluation for the combined classifier to assess the candidate solutions from diverse perspectives.
3. Third: Dimensional heterogeneity in the multiple committees established for the combined classifier. The combination strategy is essential for the ensemble method, which is majorly dependent on the design of the combined classifier. In order to create a valid combined classifier, we split the decision maker to be multiple committees and also introduced heterogeneity into these committees. By evaluating the candidate solutions from multi-dimensional points (these points are also distinct with individual classifiers), a more reliable result may be provided.

For a better understanding and also the verification of the proposed idea, the detailed designs and implementations are illustrated in the following sections. As mentioned above, we focus on the newly proposed schemes, i.e., the heterogeneity designs for the last two dimensions.

### 2.2. Heterogeneous Design for Combined Classifier and Individual Classifiers

Aiming for heterogeneity, the individual classifiers and combined classifier should follow different objective models. Since we will design multiple committees for the combined classifier, the most commonly used model in Equation (1) is reserved for the combined classifier. As to the individual classifiers, dictionary learning is employed for formulation where the sparsity is seriously considered. Therefore, whether considering the sparsity or not will be the featured heterogeneity between the combined classifier and individual classifiers.

#### 2.2.1. Dictionary Learning Model for Individual Classifiers

The dictionary learning models tries to establish a dictionary for the target signal in Equation (1) and decompose the signal with as few dictionary atoms as possible. The basic formulation is illustrated as:

(3) x=D·α

where the dictionary is defined as ***D*** = [***d***_1_,***d***_2_,…,***d****_N_*] ∈ ***R****^S^*^×*N*^, whose column ***d****_k_* ∈ ***R****^S^*^×1^ is defined as an atom. One dictionary contains *N* atoms. ***α*** ∈ ***R****^N^*^×1^ is defined as a sparsity parameter.

For a well-established model, sparsity, ***α***, has as an important role. On one hand, the dictionary, ***D***, is established based on an alternative optimization for both dictionary and sparsity. On the other hand, once the dictionary is determined, sparsity becomes a key factor for problem solving.

Therefore, based on the principles of dictionary learning, it is required to determine the dictionary, ***D*,** first. The problem is defined as:

(4) $$\underset{D,\alpha }{\min}\left\{{\left\|x-D\cdot \alpha \right\|}_{F}^{2}+\lambda g_{\alpha }\left(\alpha \right)\right\}$$

where ||•||*_F_* is the *F*-norm calculation, measuring the differences between the target and fitting in the physical sense. *λ* is the regularization parameter, indicating the proportion of sparsity in the optimization objective. *g*_•_(•) is the unified sparsity measurement function, revealing the sparsity calculation in the objective. Since both dictionary, ***D*,** and sparsity, ***α*,** are unknown variables to be solved in the model, the K-SVD algorithm is utilized to solve the alternative problem [31].

After completing the training stage, we have a feasible ***D*** for the NILM problem in a specific house. Hence, the load disaggregation problem under normal operations is a straightforward optimization, which is free of calculation burden:

(5) $$\alpha =\underset{\alpha }{\arg\min}\left\{{\left\|x-D\cdot \alpha \right\|}_{F}^{2}+\lambda g_{\alpha }\left(\alpha \right)\right\}$$

#### 2.2.2. Heterogeneous Design for the Combined Classifier

**Figure 2:**
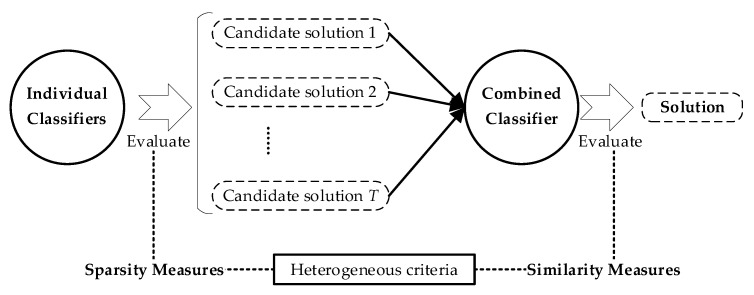
Heterogeneous design idea for the combined classifier and individual classifiers.

As seen from Equations (4) and (5), the role of sparsity may vary in the load disaggregation problem, but will always be considered in the model. However, back to the original problem in (1), sparsity is not tightly bounded. Therefore, in order to introduce the heterogeneous evaluation system, the design of the combined classifier considers the physical properties only, while the sparsity is totally ignored. The key to this idea is illustrated in Figure 2.

The architecture shown in Figure 2 provides a design sample for the heterogeneity between individual classifiers and the combined classifier. The core of this is that the evaluation criteria of individual classifiers follow sparsity measures, while those of the combined classifier follow similarity measures. The sparsity measures are calculated based on Equations (4) and (5), and diverse individual classifiers can be personalized by allocating a different regularization parameter, *λ*. The similarity measures, through which sparsity is not considered, should provide an effective and justified evaluation for the candidate solutions. Therefore, a multi-committee decision-making system is designed for the combined classifier, where different committees hold different similarity measures.

### 2.3. Heterogeneous Design for Decision-Making Committees of the Combined Classifier

**Figure 3:**
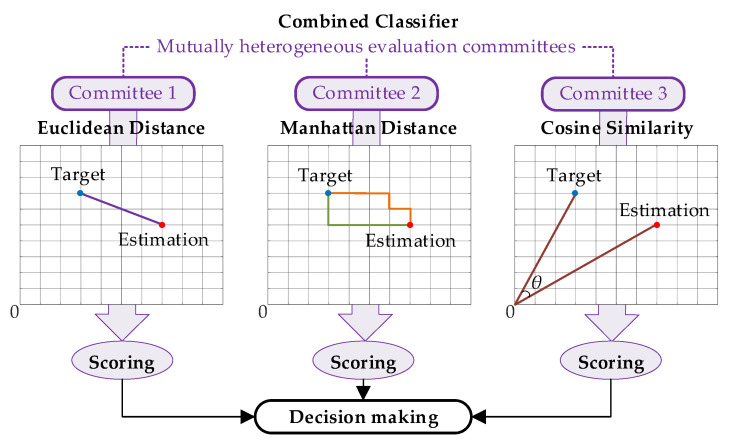
Design illustration of heterogeneous committees of the combined classifier.

Following the heterogeneous design idea for the combined classifier discussed above, diverse similarity measures should be selected for evaluation committees of the combined classifier. Among dozens of similarity measures, three commonly used measures, i.e., Euclidean distance, Manhattan distance, and cosine similarity, are selected considering the physical features of NILM. The design of the combined classifier is illustrated in Figure 3, where the physical meanings of heterogeneous committees are visualized. The basic ideas for choosing these three measures are listed below, while the rationality is demonstrated by case results:

- Euclidean distance is the most commonly used measure to evaluate the absolute distance between two points in multidimensional space. Therefore, Euclidean distance would provide an overall assessment of the differences between the estimation and target in NILM.
- Manhattan distance measures the total sum of absolute distance on each coordinate axis for a multidimensional system. Hence, Manhattan distance focuses on the fitting differences for each electric features, paying more attention to the details.
- Cosine similarity utilizes the cosine value of the angle between two vectors in multidimensional space to quantify the differences. Compared with distance measures, it is more interested in the direction as opposed to the distance or length. This measure would highlight the electric feature relevance of appliances in NILM.

#### 2.3.1. Multidimensional Space Mapping and Standardization

A vital design in similarity analysis for a multidimensional problem is how to unify the measurements of diverse dimensions together. From the view of NILM, it is essentially a trade-off problem of multi-objective fitting. This problem is quite similar to parameter tuning in many system designs, which seems insignificant but actually matters.

In practice, the unity of multiple dimensions does exist in individual classifiers, where the dictionary-learning-formulated disaggregation approach utilizes the different regularization parameters to coordinate diverse electric features together:

(6) $$\underset{\alpha }{\min}\left\{{\left\|\mathrm{norm}\left(P\right)-D_{P}\cdot \alpha \right\|}_{F}^{2}+\sum_{*\in LS}\lambda_{*}{\left\|\mathrm{norm}\left(*\right)-D_{*}\cdot \alpha \right\|}_{F}^{2}+\lambda g_{\alpha }\left(\alpha \right)\right\}$$

where norm (·) is the normalization function, ***P*** is the target signal of real power, and ***D_P_*** is the dictionary for the normalized real power analysis. D_*_ is the dictionary for the normalized electric feature of *. λ_*_ is the regularization parameter for the electric feature of *. ***LS*** is the load signature features apart from real power *P*, including reactive power, *Q*, and different orders of harmonics, *H*.

For designs with heterogeneity, the mapping and standardization for the combined classifier follows another strategy. All electric features are considered equally important, and the target is mapped to be a reference point with all dimensions equaling to unity. Consistently, the estimation is also standardized by selecting the target values as a rating base. The calculations are as follows:

(7) $$\begin{array}{l} \mathrm{Unified}\;\mathrm{Space}\;\leftarrow \;\mathrm{Mesauring}\;\mathrm{Space}\\ \;\mathrm{Target}:\;\left\{{\tilde{P}}_{\mathrm{tar}},{\tilde{Q}}_{\mathrm{tar}},...,{\tilde{H}}_{\mathrm{tar}}\right\}=\left\{1,1,...,1\right\}\leftarrow \left\{\frac{P_{\mathrm{tar}}}{P_{\mathrm{tar}}},\frac{Q_{\mathrm{tar}}}{Q_{\mathrm{tar}}},...,\frac{H_{\mathrm{tar}}}{H_{\mathrm{tar}}}\right\}\\ \;\mathrm{Estimation}\left\{{\tilde{P}}_{\mathrm{est}},{\tilde{Q}}_{\mathrm{est}},...,{\tilde{H}}_{\mathrm{est}}\right\}\leftarrow \left\{\frac{P_{\mathrm{est}}}{P_{\mathrm{tar}}},\frac{Q_{\mathrm{est}}}{Q_{\mathrm{tar}}},...,\frac{H_{\mathrm{est}}}{H_{\mathrm{tar}}}\right\} \end{array}$$

where *P*_tar_, *Q*_tar_, and *H*_tar_ are, respectively, the measured value of real power, reactive power, and harmonics, indicating the target. *P*_est_, *Q*_est_, and *H*_est_ are, respectively the estimation value of real power, reactive power, and harmonics through load disaggregation. The above variables are all related to the original electric feature space. Meanwhile, $\tilde{P}$_tar_, $\tilde{Q}$_tar_, and $\tilde{H}$_tar_ are, respectively, the standardized target of real power, reactive power, and harmonics. $\tilde{P}$_est_, $\tilde{Q}$_est_, and $\tilde{H}$_est_ are, respectively, the standardized estimation of real power, reactive power, and harmonics. These variables are considered in unified space, which are comparable.

Hence, by the above detailed designs, the combined classifier is completely heterogeneous with individual classifiers, which conforms to the proposals of this article.

#### 2.3.2. Similarity Evaluation and Scoring

With comparable multidimensional objects, it is possible to evaluate and score from different views of similarity. Following the physical meanings of the selected measures shown in Figure 3, the detailed calculations for the three committees are:

(8) $$Socre1=100\times \left(1-r_{pe}\times \frac{\sqrt{{\left({\tilde{P}}_{\mathrm{tar}}-{\tilde{P}}_{\mathrm{est}}\right)}^{2}+{\left({\tilde{Q}}_{\mathrm{tar}}-{\tilde{Q}}_{\mathrm{est}}\right)}^{2}+\cdots +{\left({\tilde{H}}_{\mathrm{tar}}-{\tilde{H}}_{\mathrm{est}}\right)}^{2}}}{\sqrt{{\tilde{P}}_{\mathrm{tar}}{}^{2}+{\tilde{Q}}_{\mathrm{tar}}{}^{2}+\cdots +{\tilde{H}}_{\mathrm{tar}}{}^{2}}}\right)$$

(9) $$Socre2=100\times \left(1-r_{pm}\times \frac{\left|{\tilde{P}}_{\mathrm{tar}}-{\tilde{P}}_{\mathrm{est}}\right|+\left|{\tilde{Q}}_{\mathrm{tar}}-{\tilde{Q}}_{\mathrm{est}}\right|+\cdots +\left|{\tilde{H}}_{\mathrm{tar}}-{\tilde{H}}_{\mathrm{est}}\right|}{\left|{\tilde{P}}_{\mathrm{tar}}\right|+\left|{\tilde{Q}}_{\mathrm{tar}}\right|+\cdots +\left|{\tilde{H}}_{\mathrm{tar}}\right|}\right)$$

(10) $$Socre3=100\times \frac{{\tilde{P}}_{\mathrm{tar}}\times {\tilde{P}}_{\mathrm{est}}+{\tilde{Q}}_{\mathrm{tar}}\times {\tilde{Q}}_{\mathrm{est}}+\cdots +{\tilde{H}}_{\mathrm{tar}}\times {\tilde{H}}_{\mathrm{est}}}{\sqrt{{\tilde{P}}_{\mathrm{tar}}{}^{2}+{\tilde{Q}}_{\mathrm{tar}}{}^{2}+\cdots +{\tilde{H}}_{\mathrm{tar}}{}^{2}}\times \sqrt{{\tilde{P}}_{\mathrm{est}}{}^{2}+{\tilde{Q}}_{\mathrm{est}}{}^{2}+\cdots +{\tilde{H}}_{\mathrm{est}}{}^{2}}}$$

where *Score*1 is the evaluated score for the candidate by the first committee of the combined classifier, following the Euclidean distance. *Score*2 is the evaluated score for the candidate by the second committee of the combined classifier, following the Manhattan distance. *Score*3 is the evaluated score for the candidate by the third committee of the combined classifier, following the Cosine similarity. *r_pe_* and *r_pm_* are, respectively, the regulation parameters for the scoring of the first and second committees. By comparing the sum of scores, the most optimal solution is determined from all candidates.

Since the candidates are generated following weighted standardization and sparsity evaluation, and selected by unified standardization and disparate measures, the decision process is totally heterogeneous. Therefore, the idea of establishing an ensemble-method-based NILM model with multidimensional heterogeneity is realized by the above implementations.

## 3. Results and Discussions

The proposed approach is tested and discussed in this section. Firstly, the evaluation metrics for NILM are presented. Then, results and discussions are provided based on simulation studies and field measurements analysis, respectively.

### 3.1. Evaluation Metrics for NILM

The most commonly used metrics evaluating the performance of NILM, including precision, sensitivity, and *F*-measure, are utilized in this section to verify the effectiveness of the proposed approach. The calculations are as follows:

(11) $$P_{s}=TP_{s}/\left(TP_{s}+FP_{s}\right)\times 100\%$$

(12) $$S_{s}=TP_{s}/\left(TP_{s}+FN_{s}\right)\times 100\%$$

(13) $$F_{s}=\left(2\times P_{s}\times S_{s}\right)/\left(P_{s}+S_{s}\right)\times 100\%$$

where *P_s_*, *S_s_*, and *F_s_* are, respectively, the precision metric, sensitivity metric, and *F*-measure metric for an appliance, *s*. *TP_s_* is the true positive disaggregation, indicating the number of detections that are correctly detected as the appliance, *s*. *FP_s_* is the false positive disaggregation, indicating the number of detections that are incorrectly detected as *s*. *FN_s_* is the false negative disaggregation, indicating the number of detections related to *s* that are incorrectly detected as other appliances.

If in the target house, all the electrical appliances form a set, Ω*_s_*. Then, the average values of all appliance metrics are utilized for the evaluation of overall NILM performance, i.e.,:

(14) $$\mathrm{Pre}=\sum_{s\in \Omega_{s}}P_{s}/N_{s},\mathrm{Sen}=\sum_{s\in \Omega_{s}}S_{s}/N_{s},F-\mathrm{mea}=\sum_{s\in \Omega_{s}}F_{s}/N_{s}$$

where Pre, Sen, and *F*-mea are, respectively, the average metric values of precision, sensitivity, and *F*-measure for an appliance set, Ω*_s_*. *N_s_* is the total number of electrical appliances in an appliance set, Ω*_s_*.

### 3.2. Studies on Low-Voltage Network Simulator

For a comprehensive investigation of the proposed idea and approach, a simulation platform, named the low-voltage network simulator (LVNS) [37], is employed in our work. Since the validation of NILM studies is one of the original motivations for developing the LVNS, it is appropriate for our extensive explorations.

A North American house, with almost twenty appliances, is simulated. The detailed information of the appliance set is shown in Table 1. As seen, all types of commonly used appliances, including ON–OFF, multi-state, repetitive mode, and transient mode, are considered in our work. Such a setup contributes to the demonstration of the validity and rationality of our study.

The proposed heterogeneity-enhanced ensemble approach is denoted as *PHA* in the following discussions. Since the individual classifier is established based on the sparse coding approach, the conventional dictionary learning approach is compared, and denoted as *CDA* [32]. In addition, the former proposal of ensemble-method-based NILM in [36], framed by the probability model, is also compared and denoted as *EPA*. Besides, in order to investigate the insights of our proposed approach, the detailed performance of individual classifiers is also recorded and analyzed. In this subsection, the individual classifiers are formed based on the feature selection bagging strategy [36], and denoted as *ICA*1, *ICA*2, *ICA*3, and *ICA*4, respectively.

#### 3.2.1. Overall NILM Performance and Comparisons

The average NILM performances by diverse approaches are shown in Table 2. As seen, by applying the ensemble strategy, the improvement of NILM performance is observed, no matter by *EPA* or *PHA*. Although the enhancement is slight, it is an important contribution to data-driven NILM research because such an improvement is achieved based on a given dataset and with the same basic disaggregation algorithm.

Comparing *PHA* with *EPA*, we find that the average performance of ensemble-method-based NILM approaches is quite similar. Nevertheless, strictly speaking, the proposed approach in this paper is slightly weaker than the probability-model-framed approach, though the margin is very small. Such results are acceptable based on the calculation burdens by these two approaches. In order to include the true solution, *EPA* conducts multiple optimization calculations for each individual classifier, which requires high computational power. However, the proposed approach in this paper requires only one calculation for each individual classifier, which is desired by the practical implementation of smart meters. The calculation burdens and statistical computation time are shown in Table 3. As seen, the optimization times for one ensemble decision by *PHA* is one-fifth of that by *EPA*. Note that the multiple optimizations by *EPA* are sequentially executed, so parallel computing methods are not applicable. Besides, the practical NILM applications are usually deployed on smart meters, so the high computation burden is not appropriate. By decreasing the optimization times, the decision time of *PHA* is correspondingly reduced to one-third. Therefore, the proposed approach shows superiority when considering both calculation performance and efficiency.

**Figure 4:**
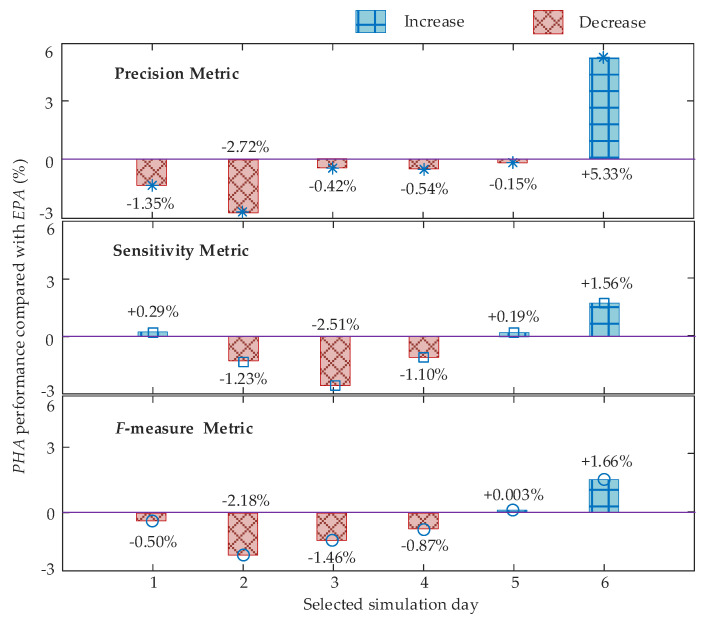
Comparison visualization of *PHA* and *EPA* in metric performance.

Besides, *PHA* does not always perform worse than *EPA*. In the simulations, six days are randomly selected, and we use the metrics results of *EPA* as references, while the relative performances of *PHA* are visualized in Figure 4. As seen, the two approaches perform similarly on day one and day five. From day two to day four, *EPA* outperforms *PHA*. However, we still have one day, i.e., day six, on which *PHA* outperforms *EPA*. Additionally, the biggest change for all metrics also happens on day six, where we have a more than 5% increase for the precision metric. Such results indicate that the proposed ensemble strategy is indeed effective and does contribute to the enhancement of NILM.

The detailed statistical disaggregation metrics for all appliances are also recorded, as shown in Table 4. The load disaggregation performances on different appliances are differed by diverse approaches. For example, *PHA* outperforms *CDA* and *EPA* for appliances HEA and LAP stably, while it shows some degradation for appliances RFR and CRTTV. Generally speaking, the proposed approach guarantees a reliable disaggregation for all appliances.

#### 3.2.2. Detailed Insights of Ensemble-Method-Based NILM

In order to reveal the effectiveness of the ensemble design for the proposed method, extensive results and discussions are provided in this subsection, focusing on the performance comparison between individual classifiers and combined classifier. The general results are shown in Table 5, while the detailed appliance results are shown in Table 6.

As seen in Table 5, by the proposed ensemble strategy, the NILM performance is improved compared with individual classifiers. The maximum enhancements of precision, sensitivity, and *F*-measure are, respectively, 6%, 11%, and 10%, while the minimum improvements are around 2%, 4%, and 4%, respectively. In general, the proposed approach shows a robust enhancement via the ensemble strategy. As seen in Table 6, once the individual classifiers perform the same, such as the results of WSH, the combined classifier also has the same results. Although the proposed approach shows a degraded performance for RFR, it successfully combines the individual classifiers for most of the other appliances, demonstrating the effectiveness of the proposed study.

Note that the heterogeneous design also plays an important role, which contributes to the improved performance in our study. In order to clarify this, an additional test is conducted. The combined classifier is redesigned with an additional decision-making committee, and the fourth committee holds the evaluation criterion similar to the individual classifiers but loses the sparsity. By doing so, the evaluation heterogeneity between individual classifiers and the combined classifier discussed in Section 2.3.1. is no longer complied strictly. This additional design is denoted as *WHA*, and the disaggregation comparisons are illustrated in Table 7.

As seen in Table 7, by ignoring the heterogeneity, the ensemble strategy is no longer effective in NILM enhancement. The performance is not only worse than our proposed method, but also the conventional dictionary learning approach, where electric features are considered all at once. Therefore, the NILM performance is highly dependent on the ensemble strategy, and our heterogeneous design is demonstrated to be effective in improving disaggregation results.

### 3.3. Studies on Field Measurement Dataset

The above comprehensive investigations on the simulation platform have verified the efficiency of the proposed approach. In order to further demonstrate the practical application capabilities of the study, a well-known public dataset, REDD [38], collected via field measurements from real houses in North America, is utilized and tested in the following discussions. Specifically, House 1 is selected for verification. The electrical appliances in this house are illustrated in Table 8.

The proposed heterogeneity-enhanced ensemble approach is still denoted as *PHA* in this subsection, as well as the compared approaches by conventional methods, i.e., *CDA* [32] and *EPA* [36]. Since the data of House 1 are low-frequency without harmonics information, the individual classifiers are generated based on the original bootstrap sampling strategy and, respectively, denoted as *ICA*1, *ICA*2, *ICA*3, and *ICA*4 in this subsection.

Table 9 provides the general results of REDD-based NILM for different approaches. As seen, by applying the ensemble strategy, the NILM performance based on field measurements are all improved. However, the ensemble design matters for the specific results. The probability-model-framed strategy in [36] achieves a higher precision, while the enhancement for sensitivity is limited, resulting in a slight improvement for *F*-measure metric. As to the proposed approach in this article, though the enhancement for the precision is not that high, there is a remarkable increase in the sensitivity metric, leading to very satisfying progress in *F*-measure.

Table 10 provides the detailed appliance disaggregation results by diverse approaches. As seen, NILM enhancement by the proposed approach is mainly due to the sensitivity metric increase for most appliances. By Table 9 and Table 10, it is observed that the heterogeneity-enhanced ensemble NILM approach is effective in load disaggregation under a field environment, even when lacking sufficient data.

For further investigations, the detailed results by individual classifiers are compared in Table 11. Because the data of field measurement are limited, the bagging strategy cannot generate highly differentiated individual classifiers. However, such deficiency is addressed by embedding the heterogeneous evaluation method into the ensemble framework. Therefore, by enhancing REDD-based NILM performance, the proposed study is verified to be an effective solution for energy monitoring.

## 4. Conclusions

In this paper, ensemble-method-based NILM studies are further investigated in terms of calculation accuracy and efficiency. For the effective utilization of the ensemble strategy in NILM, a multidimensional heterogeneity design is embedded into the NILM-oriented ensemble model. Firstly, the individual classifiers are mutually heterogeneous by following the bagging strategy. Then, the heterogeneity between individual classifiers and the combined classifier is designed by applying diverse measure calculations from two perspectives: evaluation considering sparsity or not and weighed standardization or not. Lastly, the combined classifier is also split into multiple heterogeneous decision-making committees, whose similarity evaluations are distinct from each other. Through verifications on a simulator platform and a field measurement dataset, the proposed approach is demonstrated to be able to enhance NILM performance with limited computing consumption. Besides, the heterogeneity design is effective in reinforcing the diversity requirement of the ensemble method, which shows a potential in expanding ensemble-approach-based NILM applications.

## Figures and Tables

**Table 1:** The detailed information of studied appliances in the simulation.

Phase	Appliances	Rated Power (W)	Power Factor	Operation Patterns	Code
A	ASD-based washer	320	0.45	ON–OFF, repetitive	WSH
A	Compact fluorescent lamp	60	0.9	ON–OFF	CFL
A	CRT television	200	1	Multi-state	CRTTV
A	Desktop PC	260	1	Multi-state	PC
A	Food processor	1600	1	ON–OFF	FOO
A	Furnace	600	0.84	Multi-state, transient	FUR
A	Microwave oven	1200	0.99	ON–OFF	MW
A	Regular fridge	180	0.94	ON–OFF, transient	RFR
B	Coffee maker	920	1	ON–OFF, repetitive	COF
B	Freezer	220	0.9	ON–OFF, transient	FRZR
B	Heater	1400	0.97	ON–OFF, repetitive	HEA
B	Incandescent lamp	40	1	ON–OFF	INC
B	Laptop	75	0.96	Multi-state	LAP
B	LCD computer monitor	160	0.96	Multi-state	LCD
B	LCD television	300	0.99	Multi-state	LCDTV
B	Toaster	860	1	ON–OFF	TOA
AB	Regular dryer	4000	0.88	ON–OFF, repetitive	DRY
AB	Stove	2000	0.9	ON–OFF, repetitive	STO

**Table 2:** Results comparison of the LVNS-based NILM for traditional approaches.

Metrics	*CDA*	*EPA*	*PHA*
Pre (%)	94.02	94.99	95.02
Sen (%)	85.07	87.14	86.73
*F*-mea (%)	88.30	90.23	89.72

**Table 3:** Computation burden and time comparison for ensemble approaches.

	*EPA*	*PHA*
Optimization times of individual classifiers	5	1
Optimization times for one decision	20	4
Average calculation time for one decision (s)	2.64	0.84

**Table 4:** Detailed appliance disaggregation performance of the LVNS (average value).

Appliance	*P_s_* (%)			*S_s_* (%)			*F_s_* (%)		
	*CDA*	*EPA*	*PHA*	*CDA*	*EPA*	*PHA*	*CDA*	*EPA*	*PHA*
WSH	99.78	99.66	99.66	90.09	96.42	96.42	94.61	98.01	98.01
CFL	98.79	98.62	98.37	62.43	67.14	67.46	76.23	79.76	79.89
CRTTV	92.19	92.40	90.53	66.17	70.92	62.50	76.75	80.14	73.63
PC	95.63	94.32	92.97	57.99	66.75	66.59	71.28	77.98	73.79
FOO	99.97	98.50	98.53	84.49	90.60	91.92	91.10	94.01	94.79
FUR	92.07	94.71	93.13	98.47	96.92	96.82	95.13	95.79	93.37
MW	98.71	97.94	99.85	94.68	99.40	98.27	96.61	98.65	98.95
RFR	93.14	93.87	92.58	75.21	81.52	73.30	83.02	87.02	80.47
COF	83.07	79.55	84.16	79.31	95.53	89.80	79.30	85.36	86.22
FRZR	99.95	99.99	99.96	96.43	91.14	92.49	98.15	95.23	95.90
HEA	97.42	99.98	99.98	85.09	80.69	87.89	90.60	89.30	93.42
INC	94.59	99.74	98.57	82.78	77.66	73.80	88.21	86.63	83.76
LAP	93.82	94.73	99.84	75.84	75.74	88.73	83.77	84.09	96.28
LCD	100	99.48	99.50	91.81	93.55	91.88	95.68	96.15	95.25
LCDTV	84.67	87.00	85.72	98.83	98.60	98.10	91.01	92.94	90.22
TOA	87.64	89.93	88.41	95.85	92.49	92.72	91.09	91.19	90.26
DRY	99.57	99.96	99.97	98.57	99.86	99.86	99.06	99.91	99.92
STO	81.28	89.53	88.45	97.18	93.52	92.55	87.77	91.89	91.00

**Table 5:** Results comparison of LVNS-based NILM for ensemble strategy.

Metrics	*ICA*1	*ICA*2	*ICA*3	*ICA*4	*PHA*
Pre (%)	91.18	93.34	89.16	89.04	95.02
Sen (%)	81.36	81.87	82.85	75.62	86.73
*F*-mea (%)	84.33	86.15	85.40	79.33	89.72

**Table 6:** Detailed appliance disaggregation performance of the LVNS by individual classifiers (average value).

		WSH	CFL	CRTTV	PC	FOO	FUR	MW	RFR	COF
*P_s_* (%)	*ICA*1	99.66	97.69	82.61	85.53	65.64	93.86	99.95	94.72	69.78
	*ICA*2	99.66	99.92	81.27	74.68	98.49	90.69	93.44	96.25	99.62
	*ICA*3	99.66	97.03	88.47	91.47	98.50	92.88	98.09	94.74	84.93
	*ICA*4	99.66	84.50	93.81	62.80	96.91	95.46	96.65	94.39	76.80
	*PHA*	99.66	98.37	90.53	92.97	98.53	93.13	99.85	92.58	84.16
*S_s_* (%)	*ICA*1	96.42	66.24	79.93	59.30	27.90	96.82	87.89	85.88	88.52
	*ICA*2	96.42	66.59	66.88	64.98	86.83	96.58	67.52	79.15	89.58
	*ICA*3	96.42	68.70	70.30	64.57	91.51	96.61	99.40	80.83	95.82
	*ICA*4	96.42	34.03	31.04	63.48	91.13	92.89	99.68	73.13	90.60
	*PHA*	96.42	67.46	62.50	66.59	91.92	96.82	98.27	73.30	89.80
*F_s_* (%)	*ICA*1	98.01	78.85	79.69	69.10	36.89	95.29	93.23	89.87	73.86
	*ICA*2	98.01	79.73	73.10	68.89	91.40	93.54	76.70	86.68	93.76
	*ICA*3	98.01	80.28	78.35	75.54	94.59	94.68	98.74	86.93	88.73
	*ICA*4	98.01	46.06	46.23	62.56	93.73	94.15	98.09	82.11	80.45
	*PHA*	98.01	79.89	73.63	73.79	94.79	93.37	98.95	80.47	86.22
		**FRZR**	**HEA**	**INC**	**LAP**	**LCD**	**LCDTV**	**TOA**	**DRY**	**STO**
*P_s_* (%)	*ICA*1	99.99	99.93	99.10	97.00	99.96	86.15	83.08	99.88	86.73
	*ICA*2	99.99	99.77	99.50	92.30	99.96	87.45	80.31	99.85	87.06
	*ICA*3	99.99	99.33	0	92.96	100	87.19	90.99	99.88	88.79
	*ICA*4	95.70	99.97	97.95	49.31	99.97	79.22	90.43	99.95	89.23
	*PHA*	99.96	99.98	98.57	99.84	99.50	85.72	88.41	99.97	88.45
*S_s_* (%)	*ICA*1	93.23	54.15	74.34	86.67	94.85	98.66	84.60	99.86	89.21
	*ICA*2	95.49	49.38	74.37	74.58	89.46	98.60	84.36	99.18	93.64
	*ICA*3	94.35	89.68	0	75.76	91.42	98.64	83.98	99.86	93.52
	*ICA*4	85.45	77.47	57.52	26.36	91.86	65.32	92.63	98.64	93.49
	*PHA*	92.49	87.89	73.80	88.73	91.88	98.10	92.72	99.86	92.55
*F_s_* (%)	*ICA*1	96.41	70.06	84.16	91.17	97.31	92.49	83.61	99.87	88.14
	*ICA*2	97.69	65.92	84.38	82.48	94.34	93.19	80.77	99.51	90.65
	*ICA*3	97.02	94.09	0	83.42	95.47	93.06	87.00	99.87	91.51
	*ICA*4	89.83	87.29	67.77	34.24	95.68	69.27	91.49	99.28	91.73
	*PHA*	95.90	93.42	83.76	96.28	95.25	90.22	90.26	99.92	91.00

**Table 7:** Results comparison of LVNS-based NILM considering the heterogeneity design.

**Metrics**	***WHA***	***PHA***
Pre (%)	92.97	95.02
Sen (%)	82.52	86.73
*F*-mea (%)	85.86	89.72

**Table 8:** Detailed information of studied appliances in the REDD dataset.

Appliances	Rated Power (W)	Operation Patterns	Code
Bathroom GFI	1600	ON–OFF, fluctuation	GFI
Dish washer	1000	Multi-state, complicated, and transient	DW
Dryer ^1^	5400	ON–OFF, repetitive, and fluctuation	DRY
Kitchen outlet 1	1080	ON–OFF, transient	KO1
Kitchen outlet 2	1540	ON–OFF, repetitive, and transient	KO2
Lighting 1	70	ON–OFF	LIG1
Lighting 2	80	ON–OFF	LIG2
Lighting 3	60	ON–OFF	LIG3
Microwave oven	1600	ON–OFF, fluctuation	MW
Oven 1	1650	ON–OFF, repetitive, fluctuation, and transient	OV1
Oven 2	2500	ON–OFF, repetitive, fluctuation, and transient	OV2
Regular fridge	180	ON–OFF, repetitive, and transient	RFR
Washer	600	ON–OFF, repetitive	WSH

^1^ Dryer is connected on phase A–B.

**Table 9:** General results comparison of REDD-based NILM for different approaches.

Metrics	*CDA*	*EPA*	*PHA*
Pre (%)	60.49	84.37	67.66
Sen (%)	49.17	49.62	55.60
*F*-mea (%)	49.62	53.99	56.72

**Table 10:** Detailed appliance disaggregation performance on the REDD dataset (average value).

Appliance	*P_s_* (%)			*S_s_* (%)			*F_s_* (%)		
	*CDA*	*EPA*	*PHA*	*CDA*	*EPA*	*PHA*	*CDA*	*EPA*	*PHA*
GFI	13.53	95.24	12.88	16.37	14.06	24.56	14.81	24.50	21.18
DW	99.66	99.66	99.66	78.47	61.89	78.20	87.94	76.36	87.64
DRY	91.40	92.14	91.40	89.00	98.10	89.00	90.18	95.03	90.19
KO1	0	0	0	0	0	0	0	0	0
KO2	99.23	99.01	98.25	45.07	45.97	45.97	61.99	62.79	62.64
LIG1	99.66	98.04	97.71	94.19	71.27	98.78	96.85	82.54	98.72
LIG2	99.72	98.52	98.52	14.52	14.51	16.70	25.36	25.29	28.62
LIG3	0	99.90	99.90	0	38.98	69.13	0	56.08	81.73
MW	0	100.00	0	0	20.13	0	0	33.51	0
OV1	16.96	15.78	16.97	70.95	83.73	70.96	27.38	26.55	27.38
OV2	82.20	99.50	81.79	96.52	96.04	96.04	88.79	97.74	88.34
RFR	99.81	99.40	99.21	92.23	81.38	91.69	95.87	89.49	95.30
WSH	83.61	99.60	83.27	41.93	19.06	41.76	55.85	32.00	55.63

**Table 11:** Appliance disaggregation performance on REDD by individual classifiers (average value).

		GFI	DW	DRY	KO1	KO2	LIG1	LIG2	LIG3	MW	OV1	OV2	RFR	WSH	Average
*P_s_* (%)	*ICA*1	15.18	99.66	91.4	0	98.25	97.7	98.52	0	0	16.96	81.79	99.25	83.28	60.15
	*ICA*2	15.21	99.66	92.14	0	99.01	98.04	98.52	99.90	0	15.78	99.50	99.40	99.60	70.52
	*ICA*3	15.21	99.66	92.14	0	99.01	98.04	98.52	99.90	0	15.78	99.50	99.40	99.60	70.52
	*ICA*4	15.82	99.66	92.14	0	99.01	98.04	98.52	0	0	15.96	99.50	99.40	99.60	62.90
	*PHA*	12.88	99.66	91.40	0	98.25	97.71	98.52	99.90	0	16.97	81.79	99.21	83.27	67.66
*S_s_* (%)	*ICA*1	17.52	78.21	89.00	0	45.97	72.46	14.52	0	0	70.95	96.04	91.67	41.76	47.55
	*ICA*2	13.12	61.89	98.10	0	45.97	67.96	14.51	38.98	0	83.73	96.04	81.38	19.06	47.75
	*ICA*3	13.12	61.89	98.10	0	45.97	71.27	14.51	38.98	0	83.73	96.04	81.38	19.06	48.00
	*ICA*4	13.12	61.89	98.10	0	45.97	59.23	14.39	0	0	83.73	96.04	81.38	19.06	44.07
	*PHA*	24.56	78.20	89.00	0	45.97	98.78	16.70	69.13	0	70.96	96.04	91.69	41.76	55.60
*F_s_* (%)	*ICA*1	16.27	87.64	90.18	0	62.64	83.21	25.31	0	0	27.38	88.35	95.31	55.63	48.61
	*ICA*2	14.09	76.36	95.03	0	62.79	80.27	25.29	56.08	0	26.55	97.74	89.49	32.00	50.44
	*ICA*3	14.09	76.36	95.03	0	62.79	82.54	25.29	56.08	0	26.55	97.74	89.49	32.00	50.61
	*ICA*4	14.34	76.36	95.03	0	62.79	73.85	25.12	0	0	26.81	97.74	89.49	32.00	45.66
	*PHA*	21.18	87.64	90.19	0	62.64	98.72	28.62	81.73	0	27.38	88.34	95.30	55.63	56.72

## Data Availability

The data presented in this study involve simulation data and a public dataset. The simulation platform is available in reference [37] and the public dataset is openly available in reference [38].
